# Bounded Model Checking for Hyperproperties

**DOI:** 10.1007/978-3-030-72016-2_6

**Published:** 2021-03-01

**Authors:** Tzu-Han Hsu, César Sánchez, Borzoo Bonakdarpour

**Affiliations:** 8 Eindhoven University of Technology, Eindhoven, The Netherlands; 9 Aalborg University, Aalborg East, Denmark; 10 Michigan State University, East Lansing, MI USA; 11 IMDEA Software Institute, Madrid, Spain

## Abstract

This paper introduces a bounded model checking (BMC) algorithm for *hyperproperties* expressed in HyperLTL, which — to the best of our knowledge — is the first such algorithm. Just as the classic BMC technique for LTL primarily aims at finding bugs, our approach also targets identifying counterexamples. BMC for LTL is reduced to SAT solving, because LTL describes a property via inspecting individual traces. Our BMC approach naturally reduces to QBF solving, as HyperLTL allows explicit and simultaneous quantification over multiple traces. We report on successful and efficient model checking, implemented in our tool called HyperQube, of a rich set of experiments on a variety of case studies, including security, concurrent data structures, path planning for robots, and mutation testing.

## 1 Introduction

*Hyperproperties* [10] have been shown to be a powerful framework for specifying and reasoning about important classes of requirements that were not possible with trace-based languages such as the classic temporal logics. Examples include information-flow security, consistency models in concurrent computing [6], and robustness models in cyber-physical systems [5, 35]. The temporal logic HyperLTL [9] extends LTL by allowing explicit and simultaneous quantification over execution traces, describing the property of multiple traces. For example, the security policy *observational determinism* can be specified by the following HyperLTL formula: $\forall \pi_A . \forall \pi_B . (o_{\pi_A} \leftrightarrow o_{\pi_B})\ \mathcal{W}\ \neg(i_{\pi_A} \leftrightarrow i_{\pi_B})$ which stipulates that every pair of traces $\pi_A$ and $\pi_B$ have to agree on the value of the (public) output $o$ as long as they agree on the value of the (secret) input $i$, where '$\mathcal{W}$' denotes the weak until operator.

There has been a recent surge of model checking techniques for HyperLTL specifications [9, 12, 22, 24]. These approaches employ various techniques (e.g., alternating automata, model counting, strategy synthesis, etc) to verify hyperproperties. However, they generally fall short in proposing a general push-button method to deal with identifying bugs with respect to HyperLTL formulas involving quantifier alternation. Indeed, quantifier alternation has been shown to generally elevate the complexity class of model checking HyperLTL specifications in

* This work was funded in part by the United States NSF SaTC Award 2100989, the Madrid Regional Government under project "S2018/TCS-4339 (BLOQUES-CM)", and by Spanish National Project "BOSCO (PGC2018-102210-B-100)".

different shapes of models [2,9]. For example, consider the simple Kripke structure $K$ in Fig. 1 and HyperLTL formulas $\varphi_1 = \forall\pi_A.\forall\pi_B.\Box(p_{\pi_A} \leftrightarrow p_{\pi_B})$ and $\varphi_2 = \forall\pi_A.\exists\pi_B.\Diamond\Box(p_{\pi_A} \not\leftrightarrow p_{\pi_B})$. Proving that $K \not\models \varphi_1$ (where traces for $\pi_A$ and $\pi_B$ are taken from $K$) can be reduced to building the self-composition of $K$ and applying standard LTL model checking, resulting in worst-case complexity $|K|^2$ in the size of the system. On the contrary, proving that $K \models \varphi_2$ is not as straightforward. In the worst case, this requires a subset generation to encode the existential quantifier within the Kripke structure, resulting in $|K| \cdot 2^{|K|}$ blow up. In addition, the quantification is over traces rather than states, adding to the complexity of reasoning.

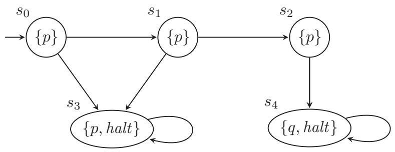

**Fig. 1:** A Kripke structure.

Following the great success of bounded model checking (BMC) for LTL specifications [8], in this paper, we propose a BMC algorithm for HyperLTL. To the best of our knowledge this is the first such algorithm. Just as BMC for LTL is reduced to SAT solving to search for a counterexample trace whose length is bounded by some integer $k$, we reduce BMC for HyperLTL to QBF solving to be able to deal with quantified counterexample traces in the input model. More formally, given a HyperLTL formula, e.g., $\varphi = \forall\pi_A.\exists\pi_B.\psi$, and a family of Kripke structures $\mathcal{K} = (K_A, K_B)$ (one per trace variable), the reduction involves three main components. First, the transition relation of $K_\pi$ (for every $\pi$) is represented by a Boolean encoding $[\![K_\pi]\!]$. Secondly, the inner LTL subformula $\psi$ is translated to a Boolean representation $[\![\psi]\!]$ in a similar fashion to the BMC unrolling technique for LTL. This way, the QBF encoding for a bound $k \geq 0$ roughly appears as:

$$[\![\mathcal{K}, \neg\varphi]\!]_k = \exists\overline{x_A}.\forall\overline{x_B}.[\![K_A]\!]_k \wedge \big([\![K_B]\!]_k \rightarrow [\![\neg\psi]\!]_k\big) \tag{1}$$

where the vector of Boolean variables $\overline{x_A}$ (respectively, $\overline{x_B}$) are used to represent the states and propositions of $K_A$ (resp. $K_B$) for steps from 0 to $k$. Formulas $[\![K_A]\!]_k$ and $[\![K_B]\!]_k$ are the unrollings $K_A$ (using $\overline{x_A}$) and $K_B$ (using $\overline{x_B}$), and $[\![\neg\psi]\!]$ (that uses both $\overline{x_A}$ and $\overline{x_B}$) is the fixpoint Boolean encoding of $\neg\psi$. The proposed technique in this paper does not incorporate a loop condition, as implementing such a condition for multiple traces is not straightforward. This, of course, comes at the cost of lack of a completeness result.

While our QBF encoding is a natural generalization of BMC for HyperLTL, the first contribution of this paper is a more refined view of how to interpret the behavior of the formula beyond the unrolling depth $k$. Consider LTL formula $\forall\pi.\Box p_\pi$. BMC for LTL attempts to find a counterexample by unrolling the model and check for satisfiability of $\exists\pi.\Diamond\neg p_\pi$ up-to bound $k$. Now consider LTL formula $\forall\pi.\Diamond p_\pi$ whose negation is $\exists\pi.\Box\neg p_\pi$. In the classic BMC, due to its *pessimistic* handling of $\Box$, the unsatisfiability of the formula cannot be established in the finite unrolling (handling these formulas requires either a looping condition or to reach the diameter of the system). This is because $\Box\neg p_\pi$ is not *sometimes finitely satisfiable* (SFS), in the terminology introduced by Havelund and Peled [27], meaning that not all satisfying traces of $\square p_\pi$ have a finite prefix that witness the satisfiability.

We propose a method that allows to interpret a wide range of outcomes of the QBF solver and relate these to the original model checking decision problem. To this end, we propose the following semantics for BMC for HyperLTL:

- *Pessimistic* semantics (like in LTL BMC) under which pending eventualities are considered to be unfulfilled. This semantics works for SFS temporal formulas and paves the way for bug hunting.
- *Optimistic* semantics considers the dual case, where pending eventualities are assumed to be fulfilled at the end of the trace. This semantics works for *sometimes finitely refutable* (SFR) formulas, and allows us to interpret unsatisfiability of QBF as proof of correctness even with bounded traces.
- *Halting* variants of the optimistic and pessimistic semantics, which allow sound and complete decision on a verdict for terminating models.

We have fully implemented our technique in the tool HyperQube. Our experimental evaluation includes a rich set of case studies, such as information-flow security, linearizability in concurrent data structures, path planning in robotic applications, and mutation testing. Our evaluation shows that our technique is effective and efficient in identifying bugs in several prominent examples. We also show that our QBF-based approach is certainly more efficient than a brute-force SAT-based approach, where universal and existential quantifiers are eliminated by combinatorial expansion to conjunctions and disjunctions. We also show that in some cases our approach can also be used as a tool for synthesis. Indeed, a witness to an existential quantifier in a HyperLTL formula is an execution path that satisfies the formula. For example, our experiments on path planning for robots showcase this feature of HyperQube.

In summary, the contributions of this paper are as follows. We (1) propose a QBF-based BMC approach for verification and falsification of HyperLTL specifications; (2) introduce complementary semantics that allow proving and disproving formulas, given a finite set of finite traces, and (3) rigorously analyze the performance of our technique by case studies from different areas of computing.

## 2 Preliminaries

### 2.1 Kripke Structures

Let $\mathsf{AP}$ be a finite set of *atomic propositions* and $\Sigma = 2^{\mathsf{AP}}$ be the *alphabet*. A *letter* is an element of $\Sigma$. A *trace* $t \in \Sigma^\omega$ over alphabet $\Sigma$ is an infinite sequence of letters: $t = t(0)t(1)t(2)\cdots$

**Definition 1.** *A* Kripke structure *is a tuple* $K = \langle S, S_{init}, \delta, L\rangle$, *where*

- $S$ *is a finite set of* states*;*
- $S_{init} \subseteq S$ *is the set of* initial states*;*
- $\delta \subseteq S \times S$ *is a* transition relation*, and*
- $L : S \to \Sigma$ *is a* labeling function *on the states of* $K$*.*

*We require that for each* $s \in S$*, there exists* $s' \in S$*, such that* $(s, s') \in \delta$*.*

Fig. 1 shows a Kripke structure, where $S_{init} = \{s_0\}$, $L(s_0) = \{p\}$, $L(s_4) = \{q, halt\}$, etc. The *size* of the Kripke structure is the number of its states. A *loop* in $K$ is a finite sequence $s(0)s(1)\cdots s(n)$, such that $(s(i), s(i+1)) \in \delta$, for all $0 \leq i < n$, and $(s(n), s(0)) \in \delta$. We call a Kripke frame *acyclic*, if the only loops are self-loops on otherwise terminal states, i.e., on states that have no other outgoing transition. Since Definition 1 does not allow terminal states, we only consider acyclic Kripke structures with such added self-loops. We also label such states by atomic proposition *halt*.

A *path* of a Kripke structure is an infinite sequence of states $s(0)s(1)\cdots \in S^\omega$, such that $s(0) \in S_{init}$, and $(s(i), s(i+1)) \in \delta$, for all $i \geq 0$. A trace of a Kripke structure is a trace $t(0)t(1)t(2)\cdots \in \Sigma^\omega$, such that there exists a path $s(0)s(1)\cdots \in S^\omega$ with $t(i) = L(s(i))$ for all $i \geq 0$. We denote by $\mathit{Traces}(K, s)$ the set of all traces of $K$ with paths that start in state $s \in S$, and use $\mathit{Traces}(K)$ as a shorthand for $\bigcup_{s \in S_{init}} \mathit{Traces}(K, s)$.

### 2.2 The Temporal Logic HyperLTL

*Syntax.* HyperLTL [9] is an extension of the linear-time temporal logic (LTL) for hyperproperties. The syntax of HyperLTL formulas is defined inductively by the following grammar:

$$\begin{aligned}\varphi &::= \exists \pi.\varphi \mid \forall \pi.\varphi \mid \phi \\ \phi &::= \mathsf{true} \mid a_\pi \mid \neg\phi \mid \phi \vee \phi \mid \phi \wedge \phi \mid \phi\,\mathcal{U}\ \phi \mid \phi\,\mathcal{R}\ \phi \mid \bigcirc\phi\end{aligned}$$

where $a \in \mathsf{AP}$ is an atomic proposition and $\pi$ is a *trace variable* from an infinite supply of variables $\mathcal{V}$. The Boolean connectives $\neg$, $\vee$, and $\wedge$ have the usual meaning, $\mathcal{U}$ is the temporal *until* operator, $\mathcal{R}$ is the temporal *release* operator, and $\bigcirc$ is the temporal *next* operator. We also consider other derived Boolean connectives, such as $\rightarrow$, and $\leftrightarrow$, and the derived temporal operators *eventually* $\Diamond\varphi \equiv \mathsf{true}\ \mathcal{U}\ \varphi$ and *globally* $\Box\varphi \equiv \neg\Diamond\neg\varphi$. Even though the set of operators presented is not minimal, we have introduced this set to uniform the treatment with the variants in Section 3. The quantified formulas $\exists\pi$ and $\forall\pi$ are read as "along some trace $\pi$" and "along all traces $\pi$", respectively. A formula is *closed* (i.e., a *sentence*) if all trace variables used in the formula are quantified. We assume, without loss of generality, that no variable is quantified twice. We use $\mathit{Vars}(\varphi)$ for the set of path variables used in formula $\varphi$.

*Semantics.* An interpretation $\mathcal{T} = \langle T_\pi \rangle_{\pi \in \mathit{Vars}(\varphi)}$ of a formula $\varphi$ consists of a tuple of sets of traces, with one set $T_\pi$ per trace variable $\pi$ in $\mathit{Vars}(\varphi)$, denoting the set of traces assigned to $\pi$. Note that we allow quantifiers to range over different models. We will use this feature in the verification of hyperproperties such as linearizability, where different quantifiers are associated with different sets of executions (in this case one for the concurrent implementation and one for the sequential implementation). That is, each set of traces comes from a Kripke structure and we use $\mathcal{K} = \langle K_\pi \rangle_{\pi \in \mathit{Vars}(\varphi)}$ to denote a *family* of Kripke structures, so $T_\pi = \mathit{Traces}(K_\pi)$ is the traces that $\pi$ can range over, which comes from $K_\pi$. Abusing notation, we write $\mathcal{T} = \mathit{Traces}(\mathcal{K})$. Note that picking a single $K$ and letting $K_\pi = K$ for all $\pi$ is a particular case, which leads to the original semantics of HyperLTL [9].

Our semantics of HyperLTL is defined with respect to a trace assignment, which is a partial map $\Pi\colon \mathit{Vars}(\varphi) \rightharpoonup \Sigma^\omega$. The assignment with the empty domain is denoted by $\Pi_\emptyset$. Given a trace assignment $\Pi$, a trace variable $\pi$, and a concrete trace $t \in \Sigma^\omega$, we denote by $\Pi[\pi \rightarrow t]$ the assignment that coincides with $\Pi$ everywhere but at $\pi$, which is mapped to trace $t$. The satisfaction of a HyperLTL formula $\varphi$ is a binary relation $\models$ that associates a formula to the models $(\mathcal{T}, \Pi, i)$ where $i \in \mathbb{Z}_{\geq 0}$ is a pointer that indicates the current evaluating position. The semantics is defined as follows:

$$
\begin{array}{lll}
(\mathcal{T}, \Pi, 0) \models \exists\pi.\ \psi & \text{iff} & \text{there is a } t \in T_\pi,\ \text{such that } (\mathcal{T}, \Pi[\pi \rightarrow t], 0) \models \psi, \\
(\mathcal{T}, \Pi, 0) \models \forall\pi.\ \psi & \text{iff} & \text{for all } t \in T_\pi,\ \text{such that } (\mathcal{T}, \Pi[\pi \rightarrow t], 0) \models \psi, \\
(\mathcal{T}, \Pi, i) \models \mathsf{true} & & \\
(\mathcal{T}, \Pi, i) \models a_\pi & \text{iff} & a \in \Pi(\pi)(i), \\
(\mathcal{T}, \Pi, i) \models \neg\psi & \text{iff} & (\mathcal{T}, \Pi, i) \not\models \psi, \\
(\mathcal{T}, \Pi, i) \models \psi_1 \vee \psi_2 & \text{iff} & (\mathcal{T}, \Pi, i) \models \psi_1 \text{ or } (\mathcal{T}, \Pi, i) \models \psi_2, \\
(\mathcal{T}, \Pi, i) \models \psi_1 \wedge \psi_2 & \text{iff} & (\mathcal{T}, \Pi, i) \models \psi_1 \text{ and } (\mathcal{T}, \Pi, i) \models \psi_2, \\
(\mathcal{T}, \Pi, i) \models \bigcirc\psi & \text{iff} & (\mathcal{T}, \Pi, i+1) \models \psi, \\
(\mathcal{T}, \Pi, i) \models \psi_1\, \mathcal{U}\, \psi_2 & \text{iff} & \text{there is a } j \geq i \text{ for which } (\mathcal{T}, \Pi, j) \models \psi_2 \text{ and} \\
 & & \quad \text{for all } k \in [i, j), (\mathcal{T}, \Pi, k) \models \psi_1, \\
(\mathcal{T}, \Pi, i) \models \psi_1\, \mathcal{R}\, \psi_2 & \text{iff} & \text{either for all } j \geq i,\ (\mathcal{T}, \Pi, j) \models \psi_2, \text{ or,} \\
 & & \quad \text{for some } j \geq i, (\mathcal{T}, \Pi, j) \models \psi_1 \text{ and} \\
 & & \quad \text{for all } k \in [i, j] : (\mathcal{T}, \Pi, k) \models \psi_2.
\end{array}
$$

This semantics is slightly different from the definition in [9], but equivalent (see [30]). We say that an interpretation $\mathcal{T}$ satisfies a sentence $\varphi$, denoted by $\mathcal{T} \models \varphi$, if $(\mathcal{T}, \Pi_\emptyset, 0) \models \varphi$. We say that a family of Kripke structures $\mathcal{K}$ satisfies a sentence $\varphi$, denoted by $\mathcal{K} \models \varphi$, if $\langle \mathit{Traces}(K_\pi) \rangle_{\pi \in \mathit{Vars}(\varphi)} \models \varphi$. When the same Kripke structure $K$ is used for all path variables we write $K \models \varphi$. For example, the Kripke structure in Fig. 1 satisfies HyperLTL formula $\varphi = \forall\pi_A.\exists\pi_B.\Diamond\Box(p_{\pi_A} \not\leftrightarrow p_{\pi_B})$.

## 3 Bounded Semantics for HyperLTL

We introduce now the bounded semantics of HyperLTL, used in Section 4 to generate queries to a QBF solver to aid solving the model checking problem.

### 3.1 Bounded Semantics

We assume the HyperLTL formula is closed and of the form $\mathbb{Q}_A\pi_A.\mathbb{Q}_B\pi_B \ldots \mathbb{Q}_Z\pi_Z.\psi$, where $\mathbb{Q} \in \{\forall, \exists\}$ and it has been converted into negation-normal form (NNF) so that the negation symbol only appears in front of atomic propositions, e.g., $\neg a_{\pi_A}$. Without loss of generality and for the sake of clarity from other numerical indices, we use roman alphabet as indices of trace variables. Thus, we assume that $\mathit{Vars}(\varphi) \subseteq \{\pi_A, \pi_B, \ldots, \pi_Z\}$. The main idea of BMC is to perform incremental exploration of the state space of the systems by unrolling the systems and the formula up-to a bound. Let $k \geq 0$ be the unrolling *bound* and let $\mathcal{T} = \langle T_A \ldots T_Z \rangle$ be a tuple of sets of traces, one per trace variable. We start by defining a satisfaction relation between HyperLTL formulas for a bounded exploration $k$ and models $(\mathcal{T}, \Pi, i)$, where $\mathcal{T}$ is the tuple of set of traces, $\Pi$ is a trace assignment mapping (as defined in Section 2), and $i \in \mathbb{Z}_{\geq 0}$ that points to the position of traces. We will define different finite satisfaction relations for general models (for $* = \mathit{pes}, \mathit{opt}, \mathit{hpes}, \mathit{hopt}$):

- $\models_k^*$, the common satisfaction relation among all semantics,
- $\models_k^{pes}$, called *pessimistic* semantics,
- $\models_k^{opt}$, called *optimistic* semantics, and
- $\models_k^{hpes}$ and $\models_k^{hopt}$, variants of $\models_k^{pes}$ and $\models_k^{opt}$ for Kripke structures that encode termination of traces (modeled as self-loops to provide infinite traces).

All these semantics coincide in the interpretation of quantifiers, Boolean connectives, and temporal operators up-to instant $k-1$, but differ in their assumptions about unseen future events after the bound of observation $k$.

**Quantifiers.** The satisfaction relation for the quantifiers is the following:

$$(\mathcal{T}, \Pi, 0) \models_k^* \exists \pi.\ \psi \quad \text{iff} \quad \text{there is a } t \in T_\pi : (\mathcal{T}, \Pi[\pi \to t], 0) \models_k^* \psi, \tag{1}$$
$$(\mathcal{T}, \Pi, 0) \models_k^* \forall \pi.\ \psi \quad \text{iff} \quad \text{for all } t \in T_\pi : (\mathcal{T}, \Pi[\pi \to t], 0) \models_k^* \psi. \tag{2}$$

**Boolean operators.** For every $i \leq k$, we have:

$$(\mathcal{T}, \Pi, i) \models_k^* \mathsf{true}, \tag{3}$$
$$(\mathcal{T}, \Pi, i) \models_k^* a_\pi \quad \text{iff} \quad a \in \Pi(\pi)(i), \tag{4}$$
$$(\mathcal{T}, \Pi, i) \models_k^* \neg a_\pi \quad \text{iff} \quad a \notin \Pi(\pi)(i), \tag{5}$$
$$(\mathcal{T}, \Pi, i) \models_k^* \psi_1 \vee \psi_2 \quad \text{iff} \quad (\mathcal{T}, \Pi, i) \models_k^* \psi_1 \text{ or } (\mathcal{T}, \Pi, i) \models_k^* \psi_2, \tag{6}$$
$$(\mathcal{T}, \Pi, i) \models_k^* \psi_1 \wedge \psi_2 \quad \text{iff} \quad (\mathcal{T}, \Pi, i) \models_k^* \psi_1 \text{ and } (\mathcal{T}, \Pi, i) \models_k^* \psi_2. \tag{7}$$

**Temporal connectives.** The case where $(i < k)$ is common between the optimistic and pessimistic semantics:

$$(\mathcal{T}, \Pi, i) \models_k^* \bigcirc \psi \quad \text{iff} \quad (\mathcal{T}, \Pi, i+1) \models_k^* \psi, \tag{8}$$
$$(\mathcal{T}, \Pi, i) \models_k^* \psi_1 \,\mathcal{U}\, \psi_2 \quad \text{iff} \quad (\mathcal{T}, \Pi, i) \models_k^* \psi_2, \text{ or } (\mathcal{T}, \Pi, i) \models_k^* \psi_1 \text{ and } (\mathcal{T}, \Pi, i+1) \models_k^* \psi_1 \mathcal{U} \psi_2, \tag{9}$$
$$(\mathcal{T}, \Pi, i) \models_k^* \psi_1 \,\mathcal{R}\, \psi_2 \quad \text{iff} \quad (\mathcal{T}, \Pi, i) \models_k^* \psi_2, \text{ and } (\mathcal{T}, \Pi, i) \models_k^* \psi_1 \text{ or } (\mathcal{T}, \Pi, i+1) \models_k^* \psi_1 \,\mathcal{R}\, \psi_2. \tag{10}$$

For $(i = k)$, in the pessimistic semantics the eventualities (including $\bigcirc$) are assumed to never be fulfilled in the future, so the current instant $k$ is the last chance:

$$
\begin{array}{llll}
(\mathcal{T},\Pi,i)\models^{pes}_k \bigcirc\psi & \text{iff} & \text{never happens}, & (P_1)\\
(\mathcal{T},\Pi,i)\models^{pes}_k \psi_1\,\mathcal{U}\,\psi_2 & \text{iff} & (\mathcal{T},\Pi,i)\models^{pes}_k \psi_2, & (P_2)\\
(\mathcal{T},\Pi,i)\models^{pes}_k \psi_1\,\mathcal{R}\,\psi_2 & \text{iff} & (\mathcal{T},\Pi,i)\models^{pes}_k \psi_1\wedge\psi_2. & (P_3)
\end{array}
$$

On the other hand, in the optimistic semantics the eventualities are assumed to be fulfilled in the future:

$$
\begin{array}{llll}
(\mathcal{T},\Pi,i)\models^{opt}_k \bigcirc\psi & \text{iff} & \text{always happens}, & (O_1)\\
(\mathcal{T},\Pi,i)\models^{opt}_k \psi_1\,\mathcal{U}\,\psi_2 & \text{iff} & (\mathcal{T},\Pi,i)\models^{opt}_k \psi_1\vee\psi_2, & (O_2)\\
(\mathcal{T},\Pi,i)\models^{opt}_k \psi_1\,\mathcal{R}\,\psi_2 & \text{iff} & (\mathcal{T},\Pi,i)\models^{opt}_k \psi_2. & (O_3)
\end{array}
$$

To capture the halting semantics, we use the predicate *halt* that is true if the state corresponds to a halting state (self-loop), and define $halted \stackrel{\text{def}}{=} \bigwedge_{\pi\, Vars(\varphi)} halt_\pi$ which holds whenever all traces have halted (and their final state will be repeated ad infinitum). Then, the halted semantics of the temporal case for $i = k$ in the pessimistic case consider the halting case to infer the actual value of the temporal operators on the (now fully known) trace:

$$
\begin{array}{llll}
(\mathcal{T},\Pi,i)\models^{hpes}_k \bigcirc\psi & \text{iff} & (\mathcal{T},\Pi,i)\models^{*}_k halted \text{ and } (\mathcal{T},\Pi,i)\models^{hpes}_k \psi & (HP_1)\\
(\mathcal{T},\Pi,i)\models^{hpes}_k \psi_1\,\mathcal{U}\,\psi_2 & \text{iff} & (\mathcal{T},\Pi,i)\models^{hpes}_k \psi_2 & (HP_2)\\
(\mathcal{T},\Pi,i)\models^{hpes}_k \psi_1\,\mathcal{R}\,\psi_2 & \text{iff} & (\mathcal{T},\Pi,i)\models^{hpes}_k \psi_1\wedge\psi_2, \text{ or} & \\
 & & (\mathcal{T},\Pi,i)\models^{*}_k halted \text{ and } (\mathcal{T},\Pi,i)\models^{hpes}_k \psi_2 & (HP_3)
\end{array}
$$

Dually, in the halting optimistic case:

$$
\begin{array}{llll}
(\mathcal{T},\Pi,i)\models^{hopt}_k \bigcirc\psi & \text{iff} & (\mathcal{T},\Pi,i)\not\models^{*}_k halted \text{ or } (\mathcal{T},\Pi,i)\models^{hopt}_k \psi & (HO_1)\\
(\mathcal{T},\Pi,i)\models^{hopt}_k \psi_1\,\mathcal{U}\,\psi_2 & \text{iff} & (\mathcal{T},\Pi,i)\models^{hopt}_k \psi_2, \text{ or} & \\
 & & (\mathcal{T},\Pi,i)\not\models^{*}_k halted \text{ and } (\mathcal{T},\Pi,i)\models^{hopt}_k \psi_1 & (HO_2)\\
(\mathcal{T},\Pi,i)\models^{hopt}_k \psi_1\,\mathcal{R}\,\psi_2 & \text{iff} & (\mathcal{T},\Pi,i)\models^{hpes}_k \psi_2 & (HO_3)
\end{array}
$$

**Complete semantics.** We are now ready to define the four semantics:
- Pessimistic semantics: $\models^{pes}_k$ use rules (1)-(10) and $(P_1)$-$(P_3)$.
- Optimistic semantics: $\models^{opt}_k$ use rules (1)-(10) and $(O_1)$-$(O_3)$.
- Halting pessimistic semantics: $\models^{hpes}_k$ use rules (1)-(10) and $(HP_1)$-$(HP_3)$.
- Halting optimistic semantics: $\models^{hopt}_k$ use rules (1)-(10) and $(HO_1)$-$(HO_3)$.

### 3.2 The Logical Relation between Different Semantics

Observe that the pessimistic semantics is the semantics in the traditional BMC for LTL.In the pessimistic semantics a formula is declared false unless it is witnessed to be true within the bound explored. In other words, formulas can only get "truer" with more information obtained by a longer unrolling. Dually, the optimistic semantics considers a formula true unless there is evidence within the bounded exploration on the contrary. Therefore, formulas only get "falser" with further unrolling. For example, formula $\square p$ always evaluates to false in the pessimistic semantics. In the optimistic semantics, it evaluates to true up-to bound

$k$ if $p$ holds in all states of the trace up-to and including $k$. However, if the formula evaluates to false at some point before $k$, then it evaluates to false for all $j \geq k$. The following lemma formalizes this intuition in HyperLTL.

**Lemma 1.** *Let $k \leq j$. Then,*
1. *If $(\mathcal{T}, \Pi, 0) \models_k^{pes} \varphi$, then $(\mathcal{T}, \Pi, 0) \models_j^{pes} \varphi$.*
2. *If $(\mathcal{T}, \Pi, 0) \not\models_k^{opt} \varphi$, then $(\mathcal{T}, \Pi, 0) \not\models_j^{opt} \varphi$.*
3. *If $(\mathcal{T}, \Pi, 0) \models_k^{hpes} \varphi$, then $(\mathcal{T}, \Pi, 0) \models_j^{hpes} \varphi$.*
4. *If $(\mathcal{T}, \Pi, 0) \not\models_k^{hopt} \varphi$, then $(\mathcal{T}, \Pi, 0) \not\models_j^{hopt} \varphi$.*

In turn, the verdict obtained from the exploration up-to $k$ can (in some cases) be used to infer the verdict of the model checking problem. As in classical BMC, if the pessimistic semantics find a model, then it is indeed a model. Dually, if our optimistic semantics fail to find a model, then there is no model. The next lemma formally captures this intuition.

**Lemma 2 (Infinite inference).** *The following hold for every $k$,*
1. *If $(\mathcal{T}, \Pi, 0) \models_k^{pes} \varphi$, then $(\mathcal{T}, \Pi, 0) \models \varphi$.*
2. *If $(\mathcal{T}, \Pi, 0) \not\models_k^{opt} \varphi$, then $(\mathcal{T}, \Pi, 0) \not\models \varphi$.*
3. *If $(\mathcal{T}, \Pi, 0) \models_k^{hpes} \varphi$, then $(\mathcal{T}, \Pi, 0) \models \varphi$.*
4. *If $(\mathcal{T}, \Pi, 0) \not\models_k^{hopt} \varphi$, then $(\mathcal{T}, \Pi, 0) \not\models \varphi$.*

*Example 1.* Consider the Kripke structure in Fig. 1, bound $k = 3$, and formula $\varphi_1 = \forall \pi_A.\exists \pi_B.\big((p_{\pi_A} \not\leftrightarrow p_{\pi_B})\, \mathcal{R}\, \neg q_{\pi_A}\big)$. It is easy to see that instantiating $\pi_A$ with trace $s_0 s_1 s_2 s_4$ falsifies $\varphi_1$ in the pessimistic semantics. By Lemma 2, this counterexample shows that the Kripke structure is a model of $\neg\varphi_1$ in the infinite semantics as well. That is, $K \models_3^{pes} \neg\varphi_1$ and, hence, $K \models \neg\varphi_1$, so $K \not\models \varphi_1$.

Consider again the same Kripke structure, bound $k = 3$, and formula $\varphi_2 = \forall \pi_A.\exists \pi_B.\Diamond(p_{\pi_A} \leftrightarrow q_{\pi_B})$. To disprove $\varphi_2$, we need to find a trace $\pi_A$ such that for all other $\pi_B$, proposition $q$ in $\pi_B$ always disagrees with $p$ in $\pi_A$. It is straightforward to observe that such a trace $\pi_A$ does not exist. By Lemma 2, proving the formula is not satisfiable up-to bound 3 in the optimistic semantics implies that $K$ is not a model of $\neg\varphi_2$ in the infinite semantics. That is, $K \not\models_3^{opt} \neg\varphi_2$ implies $K \not\models \neg\varphi_2$. Hence, we conclude $K \models \varphi_2$.

Consider again the same Kripke structure which has two terminating states, $s_3$ and $s_4$, labeled by atomic proposition *halt* with only a self-loop. Let $k = 3$, and $\varphi_3 = \forall \pi_A.\exists \pi_B.(\neg q_{\pi_B}\, \mathcal{U}\, \neg p_{\pi_A})$. Instantiating $\pi_A$ by trace $s_0 s_1 s_3$, which is of the form $\{p\}^\omega$ satisfies $\neg\varphi_3$. By Lemma 2, the fulfillment of formula implies that in infinite semantics it will be fulfilled as well. That is, $K \models_3^{hpes} \neg\varphi_3$ implies $K \models \neg\varphi_3$. Hence, $K \not\models \varphi_3$.

Consider again the same Kripke structure with halting states and formula $\varphi_4 = \forall \pi_A.\exists \pi_B.\Diamond\Box(p_{\pi_A} \not\leftrightarrow p_{\pi_B})$. A counterexample is an instantiation of $\pi_A$ such that for all $\pi_B$, both traces will always eventually agree on $p$. Trace $s_0 s_1 s_2 s_4$, which is of the form $\{p\}\{p\}\{p\}\{q, \mathit{halt}\}^\omega$ with $k = 3$. This trace never agrees with a trace that ends in state $s_3$ (which is of the form $\{p\}^\omega$) and vice versa. By Lemma 2, the absence of counterexample up-to bound 3 in the halting optimistic semantics implies that $K$ is not a model of $\neg\varphi_4$ in the infinite semantics. That is, $K \not\models_3^{hopt} \neg\varphi_4$ implies $K \not\models \neg\varphi_4$. Hence, we conclude $K \models \varphi_4$. □

## 4 Reducing BMC to QBF Solving

Given a family of Kripke structures $\mathcal{K}$, a HyperLTL formula $\varphi$, and bound $k \geq 0$, our goal is to construct a QBF formula $[\![\mathcal{K}, \varphi]\!]_k$ whose satisfiability can be used to infer whether or not $\mathcal{K} \models \varphi$.

In the following paragraphs, we first describe how to encode the model and the formula, and then how to combine the two to generate the QBF query. We will illustrate the constructions using formula $\varphi_1$ in Example 1 in Section 3, whose negation is $\exists\pi_A.\forall\pi_B.\neg\psi$ with $\neg\psi = (p_{\pi_A} \leftrightarrow p_{\pi_B})\,\mathcal{U}\,q_{\pi_A}$.

*Encoding the models.* The unrolling of the transition relation of a Kripke structure $K_A = \langle S, S_{init}, \delta, L\rangle$ up to bound $k$ is analogous to the BMC encoding for LTL [8]. First, note that the state space $S$ can be encoded with a (logarithmic) number of bits in $|S|$. We introduce additional variables $n_0, n_1, \ldots$ to encode the state of the Kripke structure and use $\mathsf{AP}^* = \mathsf{AP} \cup \{n_0, n_1, \ldots\}$ for the extended alphabet that includes the encoding of $S$. In this manner, the set of initial states of a Kripke structure is a Boolean formula over $\mathsf{AP}^*$. For example, for the Kripke structure $K_A$ in Fig. 1 the set of initial states (in this case $S_{init} = \{s_0\}$) corresponds to the following Boolean formula:

$$I_A := (\neg n_0 \wedge \neg n_1 \wedge \neg n_2) \wedge p \wedge \neg q \wedge \neg halt$$

assuming that $(\neg n_0 \wedge \neg n_1 \wedge \neg n_2)$ represents state $s_0$ (we need three bits to encode five states.) Similarly, $R_A$ is a binary relation that encodes the transition relation $\delta$ of $K_A$ (representing the relation between a state and its successor). The encoding into QBF works by introducing fresh Boolean variables (a new copy of $\mathsf{AP}^*$ for each Kripke structure $K_A$ and position), and then producing a Boolean formula that encodes the unrolling up-to $k$. We use $x_A^i$ for the set of fresh copies of the variables $\mathsf{AP}^*$ of $K_A$ corresponding to position $i \in [0, k]$. Therefore, there are $k|x_A| = k|\mathsf{AP}_A^*|$ Boolean variables to represent the unrolling of $K_A$. We use $I_A(x)$ for the Boolean formula (using variables from $x$) that encodes the initial states, and $R_A(x, x')$ (for two copies of the variables $x$ and $x'$) for the Boolean formula whether $x'$ encodes a successor states of $x$. For example, for $k = 3$, we unroll the transition relation up-to 3 as follows,

$$[\![K_A]\!]_3 = I_A(x_A^0) \wedge R_A(x_A^0, x_A^1) \wedge R(x_A^1, x_A^2) \wedge R(x_A^2, x_A^3)$$

which is the Boolean formula representing valid traces of length 4, using four copies of the variables $\mathsf{AP}_A^*$ that represent the Kripke structure $K_A$.

*Encoding the inner LTL formula.* The idea of the construction of the inner LTL formula is analogous to standard BMC as well, except for the choice of different semantics described in Section 3. In particular, we introduce the following inductive construction and define four different unrollings for a given $k$: $[\![\cdot]\!]_{i,k}^{pes}$, $[\![\cdot]\!]_{i,k}^{opt}$, $[\![\cdot]\!]_{i,k}^{hpes}$, and $[\![\cdot]\!]_{i,k}^{hopt}$.

– **Inductive Case**: Since the semantics only differ on the temporal operators at the end of the unrolling, the inductive case is common to all unrollings and we use $[\![\cdot]\!]^*_{i,k}$ to mean any of the choices of semantic (for $* =$ *pes*, *opt*, *hpes*, *hopt*). For all $i \leq k$:

$$
\begin{array}{lcl}
[\![p_\pi]\!]^*_{i,k} & := & p^i_\pi \\
[\![\neg p_\pi]\!]^*_{i,k} & := & \neg p^i_\pi \\
[\![\psi_1 \vee \psi_2]\!]^*_{i,k} & := & [\![\psi_1]\!]^*_{i,k} \vee [\![\psi_2]\!]^*_{i,k} \\
[\![\psi_1 \wedge \psi_2]\!]^*_{i,k} & := & [\![\psi_1]\!]^*_{i,k} \wedge [\![\psi_2]\!]^*_{i,k} \\
[\![\psi_1 \ \mathcal{U} \ \psi_2]\!]^*_{i,k} & := & [\![\psi_2]\!]^*_{i,k} \ \vee \ \Big([\![\psi_1]\!]^*_{i,k} \wedge [\![\psi_1 \ \mathcal{U} \ \psi_2]\!]^*_{i+1,k}\Big) \\
[\![\psi_1 \ \mathcal{R} \ \psi_2]\!]^*_{i,k} & := & [\![\psi_2]\!]^*_{i,k} \ \wedge \ \Big([\![\psi_1]\!]^*_{i,k} \vee [\![\psi_1 \ \mathcal{R} \ \psi_2]\!]^*_{i+1,k}\Big) \\
[\![\bigcirc \psi]\!]^*_{i,k} & := & [\![\psi]\!]^*_{i+1,k}
\end{array}
$$

Note that, for a given path variable $\pi_A$, the atom $p^i_{\pi_A}$ that results from $[\![p_{\pi_A}]\!]^*_{i,k}$ is one of the Boolean variables in $x^i_A$.

– For the **base case**, the formula generated is different depending on the intended semantics:

$$
\begin{array}{ll}
[\![\psi]\!]^{pes}_{k+1,k} := \mathsf{false} & [\![\psi]\!]^{opt}_{k+1,k} := \mathsf{true} \\
[\![\psi]\!]^{hpes}_{k+1,k} := [\![halted]\!]^{hpes}_{k,k} \wedge [\![\psi]\!]^{hpes}_{k,k} & [\![\psi]\!]^{hopt}_{k+1,k} := [\![halted]\!]^{hopt}_{k,k} \rightarrow [\![\psi]\!]^{hopt}_{k,k}
\end{array}
$$

Note that the base case defines the value to be assumed for the formula after the end $k$ of the unrolling, which is spawned in the temporal operators in the inductive case at $k$. The pessimistic semantics assume the formula to be false, and the optimistic semantics assume the formula to be true. The halting cases consider the case at which the traces have halted (using in this case the evaluation at $k$) and using the unhalting choice otherwise.

*Example 2.* Consider again the formula $\neg\psi = (p_{\pi_A} \leftrightarrow p_{\pi_B}) \, \mathcal{U} \, q_{\pi_A}$. Using the pessimistic semantics $[\![\neg\psi]\!]^{pes}_{0,3}$ with three steps is

$$
q^0_{\pi_A} \vee \Big((p^0_{\pi_A} \leftrightarrow p^0_{\pi_B}) \wedge \Big(q^1_{\pi_A} \vee \Big((p^1_{\pi_A} \leftrightarrow p^1_{\pi_B}) \wedge \Big(q^2_{\pi_A} \vee (p^2_{\pi_A} \leftrightarrow p^2_{\pi_B}) \wedge q^3_{\pi_A}\Big)\Big)\Big)\Big).
$$

In this encoding, the collection $x^2_A$, contains all variables of $\mathsf{AP}^*$ of $K_A$ (that is $\{p^2_{\pi_A}, q^2_{\pi_A}, \ldots\}$) connecting to the corresponding valuation for $p_{\pi_A}$ in the trace of $K_A$ at step 2 in the unrolling of $K_A$. In other words, the formula $[\![\neg\psi]\!]^{pes}_{0,3}$ uses variables from $x^0_A, x^1_A, x^2_A, x^3_A$ and $x^0_B, x^1_B, x^2_B, x^3_B$ (that is, from $\overline{x_A}$ and $\overline{x_B}$). □

*Combining the encodings.* Now, let $\varphi$ be a HyperLTL formula of the form $\varphi = \mathbb{Q}_A \pi_A . \mathbb{Q}_B \pi_B . \ldots . \mathbb{Q}_Z \pi_Z . \psi$ and $\mathcal{K} = \langle K_A, K_B, \ldots, K_Z \rangle$. Combining all the components, the encoding of the HyperLTL BMC problem in QBF is the following (for $* =$ *pes*, *opt*, *hpes*, *hopt*):

$$
[\![\mathcal{K}, \varphi]\!]^*_k = \mathbb{Q}_A \overline{x_A} . \mathbb{Q}_B \overline{x_B} \cdots . \mathbb{Q}_Z \overline{x_Z} \Big( [\![K_A]\!]_k \circ_A [\![K_B]\!]_k \circ_B \cdots [\![K_Z]\!]_k \circ_Z [\![\psi]\!]^*_{0,k} \Big)
$$

where $[\![\psi]\!]^*_{0,k}$ is the choice of semantics, $\circ_j = \wedge$ if $\mathbb{Q}_j = \exists$, and $\circ_j = \rightarrow$ if $\mathbb{Q}_j = \forall$, for $j \in \mathit{Vars}(\varphi)$.

*Example 3.* Consider again Example 2. To combine the model description with the encoding of the HyperLTL formula, we use two identical copies of the given Kripke structure to represent different paths $\pi_A$ and $\pi_B$ on the model, denoted as $K_A$ and $K_B$. The final resulting formula is:

$$[\![\mathcal{K}, \neg\varphi]\!]_3 := \exists \overline{x_A}.\forall \overline{x_B}.\big([\![K_A]\!]_3 \wedge ([\![K_B]\!]_3 \rightarrow [\![\neg\varphi]\!]_{0,3}^{pes})\big)$$

The sequence of assignments $(\neg n_2, \neg n_1, \neg n_0, p, \neg q, \neg halt)^0$ $(\neg n_2, \neg n_1, n_0, p, \neg q, \neg halt)^1$ $(\neg n_2, n_1, \neg n_0, p, \neg q, \neg halt)^2$ $(n_2, \neg n_1, \neg n_0, \neg p, q, halt)^3$ on $K_A$, corresponding to the path $s_0 s_1 s_2 s_4$, satisfies $[\![\neg\varphi]\!]_{0,3}^{pes}$ for all traces on $K_B$. The satisfaction result shows that $[\![\mathcal{K}, \neg\varphi]\!]_3^{pes}$ is true, indicating that a witness of violation is found. Theorem 1, by a successful detection of a counterexample witness, and the use of the pessimistic semantics, allows to conclude that $\mathcal{K} \not\models \varphi$. □

The main result of this section is Theorem 1 that connects the output of the solver to the original model checking problem. We first show an auxiliary lemma.

**Lemma 3.** *Let $\varphi$ be a closed HyperLTL formula and $\mathcal{T} = Traces(\mathcal{K})$ be an interpretation. For $* = pes, opt, hpes, hopt$, it holds that*

$$[\![\mathcal{K}, \varphi]\!]_k^* \text{ is satisfiable if and only if } (\mathcal{T}, \Pi_\emptyset, 0) \models_k^* \varphi.$$

*Proof (sketch).* The proof proceeds in two steps. First, let $\psi$ be the largest quantifier-free sub-formula of $\varphi$. Then, every tuple of traces of length $k$ (one for each $\pi$) is in one-to-one correspondence with the collection of variables $p_\pi^i$, that satisfies that the tuple is a model of $\psi$ (in the choice semantics) if and only if the corresponding assignment makes $[\![\psi]\!]_0^*$. Then, the second part shows inductively in the stack of quantifiers that each subformula obtained by adding a quantifier is satisfiable if and only if the semantics hold. □

Lemma 3, together with Lemma 2, allows to infer the outcome of the model checking problem from satisfying (or unsatisfying) instances of QBF queries, summarized in the following theorem.

**Theorem 1.** *Let $\varphi$ be a HyperLTL formula. Then,*
1. *For $* = pes, hpes$, if $[\![\mathcal{K}, \neg\varphi]\!]_k^*$ is satisfiable, then $\mathcal{K} \not\models \varphi$.*
2. *For $* = opt, hopt$, if $[\![\mathcal{K}, \neg\varphi]\!]_k^*$ is unsatisfiable, then $\mathcal{K} \models \varphi$.*

Table 1 illustrates what Theorem 1 allows to soundly conclude from the output of the QBF solver about the model checking problem of formulas from Example 1 in Section 3.

## 5 Evaluation and Case Studies

We now evaluate our approach by a rich set of case studies on information-flow security, concurrent data structures, path planning for robots, and mutation testing. In this section, we will refer to each property in HyperLTL as in Table 2.

| | | Semantics | | |
|---|---|---|---|---|
| **Formula** | **Bound** | *pessimistic* | *optimistic* | *halting* |
| $\varphi_1$ | $k = 2$ | UNSAT (inconclusive) | SAT (inconclusive) | UNSAT (inconclusive) |
| | $k = 3$ | SAT (***counterexample***) | SAT (inconclusive) | UNSAT (inconclusive) |
| $\varphi_2$ | $k = 2$ | UNSAT (inconclusive) | SAT (inconclusive) | UNSAT (inconclusive) |
| | $k = 3$ | UNSAT (inconclusive) | UNSAT (***proved***) | UNSAT (inconclusive) |
| $\varphi_3$ | $k = 2$ | UNSAT (inconclusive) | UNSAT (inconclusive) | non-halted (inconclusive) |
| | $k = 3$ | UNSAT (inconclusive) | UNSAT (inconclusive) | halted (***counterexample***) |
| $\varphi_4$ | $k = 2$ | UNSAT (inconclusive) | UNSAT (inconclusive) | non-halted (inconclusive) |
| | $k = 3$ | UNSAT (inconclusive) | UNSAT (inconclusive) | halted (***proved***) |

**Table 1:** Comparison of Properties with Different Semantics

We have implemented the technique described in Section 4 in our tool HyperQube. Given a transition relation, the tool automatically unfolds it up to $k \geq 0$ by a home-grown procedure written in Ocaml, called genqbf. Given the choice of the semantics (pessimistic, optimistic, and halting variants) the unfolded transition relation is combined with the QBF encoding of the input HyperLTL formula to form a complete QBF instance which is then fed to the QBF solver QuAbS [28]. All experiments in this section are run on an iMac desktop with Intel i7 CPU @3.4 GHz and 32 GB of RAM. A full description of the systems and formulas used can be accessed in the longer version of this paper [30].

**Case Study 1: Symmetry in Lamport's Bakery algorithm [12].** Symmetry states that no specific process has special privileges in terms of a faster access to the critical section (see different symmetry formulas in Table 2). In these formulas, each process $P_n$ has a program counter denoted by $pc(P_n)$, *select* indicates which process is selected to process next, *pause* if both processes are not selected, *sym_break* is which process is selected after a tie, and $\mathsf{sym}(select_{\pi_A}, select_{\pi_B})$ indicates if two traces are selecting two opposite processes. The Bakery algorithm does not satisfy symmetry (i.e. $\varphi_{\mathsf{sym}_1}$), because when two or more processes are trying to enter the critical section with the same ticket number, the algorithm always gives priority to the process with the smaller process ID. HyperQube returns SAT using the pessimistic semantics, indicating that there exists a counterexample in the form of a falsifying witness to $\pi_A$ in formula $\varphi_{\mathsf{sym}_1}$. Table 3 includes our result on other symmetry formulas presented in Table 2.

**Case Study 2: Linearizability in SNARK [14].** SNARK implements a concurrent double-ended queue using double-compare-and-swap (DCAS) and a doubly linked-list that stores values in each node. *Linearizability* [29] requires that any *history* of execution of a concurrent data structure (i.e., sequence of *invocation* and *response* by different threads) matches some sequential order of invocations and responses (see formula $\varphi_{\mathsf{lin}}$ in Table 2). SNARK is known to have two linearizability bugs and HyperQube returns SAT using the pessimistic semantics, identifying both bugs as two counterexamples. The bugs we identified are precisely the same as the ones reported in [14].

| Property | Property in HyperLTL |
|---|---|
| Symmetry | $\varphi_{\mathsf{S1}} = \forall \pi_A.\forall \pi_B.\big(\neg \mathsf{sym}(select_{\pi_A}, select_{\pi_B}) \vee \neg(pause_{\pi_A} = pause_{\pi_B})\big)\mathcal{R}$ $\big((pc(P_0)_{\pi_A} = pc(P_1)_{\pi_B}) \wedge (pc(P_1)_{\pi_A} = pc(P_0)_{\pi_B})\big)$ |
| | $\varphi_{\mathsf{S2}} = \forall \pi_A.\forall \pi_B.\big(\neg \mathsf{sym}(select_{\pi_A}, select_{\pi_B}) \vee \neg(pause_{\pi_A} = pause_{\pi_B}) \vee$ $\neg(select_{\pi_A} < 3) \vee \neg(select_{\pi_B} < 3)\big)\mathcal{R}$ $\big((pc(P_0)_{\pi_A} = pc(P_1)_{\pi_B}) \wedge (pc(P_1)_{\pi_A} = pc(P_0)_{\pi_B})\big)$ |
| | $\varphi_{\mathsf{S3}} = \forall \pi_A.\forall \pi_B.\big(\neg \mathsf{sym}(select_{\pi_A}, select_{\pi_B}) \vee \neg(pause_{\pi_A} = pause_{\pi_B}) \vee$ $\neg(select_{\pi_A} < 3) \vee \neg(select_{\pi_B} < 3) \vee$ $\neg \mathsf{sym}(sym\_break_{\pi_A}, sym\_break_{\pi_B})\big)\mathcal{R}$ $\big((pc(P_0)_{\pi_A} = pc(P_1)_{\pi_B}) \wedge (pc(P_1)_{\pi_A} = pc(P_0)_{\pi_B})\big)$ |
| | $\varphi_{\mathsf{sym}_1} = \forall \pi_A.\exists \pi_B.\square \mathsf{sym}(select_{\pi_A}, select_{\pi_B}) \wedge (pause_{\pi_A} = pause_{\pi_B}) \wedge$ $(pc(P_0)_{\pi_A} = pc(P_1)_{\pi_B}) \wedge (pc(P_1)_{\pi_A} = pc(P_0)_{\pi_B})$ |
| | $\varphi_{\mathsf{sym}_2} = \forall \pi_A.\exists \pi_B.\square \mathsf{sym}(select_{\pi_A}, select_{\pi_B}) \wedge (pause_{\pi_A} = pause_{\pi_B}) \wedge$ $(select_{\pi_A} < 3) \wedge (select_{\pi_B} < 3) \wedge$ $(pc(P_0)_{\pi_A} = pc(P_1)_{\pi_B}) \wedge (pc(P_1)_{\pi_A} = pc(P_0)_{\pi_B})$ |
| Linearizability | $\varphi_{\mathsf{lin}} = \forall \pi_A.\exists \pi_B.\ \square(history_{\pi_A} \leftrightarrow history_{\pi_B})$ |
| NI | $\varphi_{\mathsf{NI}} = \forall \pi_A.\exists \pi_B.\big(PIN_{\pi_A} \neq PIN_{\pi_B}\big) \wedge \big((\neg halt_{\pi_A} \vee \neg halt_{\pi_B})$ $\mathcal{U}\ \big((halt_{\pi_A} \wedge halt_{\pi_B}) \wedge (Result_{\pi_A} = Result_{\pi_B})\big)\big)$ |
| Fairness | $\varphi_{\mathsf{fair}} = \exists \pi_A.\forall \pi_B.\ (\diamondsuit m_{\pi_A}) \wedge (\diamondsuit NRR_{\pi_A}) \wedge (\diamondsuit NRO_{\pi_A}) \wedge$ $\big((\square \bigwedge_{act \in Act_P} act_{\pi_A} \leftrightarrow act_{\pi_B}) \rightarrow ((\diamondsuit NRR_{\pi_B}) \leftrightarrow (\diamondsuit NRO_{\pi_B}))\big) \wedge$ $\big((\square \bigwedge_{act \in Act_Q} act_{\pi_A} \leftrightarrow act_{\pi_B}) \rightarrow ((\diamondsuit NRR_{\pi_B}) \leftrightarrow (\diamondsuit NRO_{\pi_B}))\big)$ |
| Path Planning | $\varphi_{\mathsf{sp}} = \exists \pi_A.\forall \pi_B.(\neg goal_{\pi_B}\ \mathcal{U}\ goal_{\pi_A})$ |
| | $\varphi_{\mathsf{rb}} = \exists \pi_A.\forall \pi_B.\ (strategy_{\pi_B} \leftrightarrow strategy_{\pi_A})\,\mathcal{U}\,(goal_{\pi_A} \wedge goal_{\pi_B})$ |
| Mutant | $\varphi_{\mathsf{mut}} = \exists \pi_A.\forall \pi_B(\mathsf{mut}_{\pi_A} \wedge \neg \mathsf{mut}_{\pi_B}) \wedge \big((in_{\pi_A} \leftrightarrow in_{\pi_B})\ \mathcal{U}\ (out_{\pi_A} \not\leftrightarrow out_{\pi_B})\big)$ |

**Table 2:** Hyperproperties investigated in case studies.

**Case Study 3: Non-interference in multi-threaded Programs.** *Non-interference* [25] states that low-security variables are independent from the high-security variables, thus preserving secure information flow. We consider the concurrent program example in [32], where *PIN* is high security input and *Result* is low security output. HyperQube returns SAT in the halting pessimistic semantics, indicating that there is a trace that we can detect the difference of a high-variable by observing a low variable, that is, violating non-interference. We also verified the correctness of a fix to this algorithm, proposed in [32] as well. HyperQube uses the UNSAT results from the solver (with halting optimistic semantics) to infer the absence of violation, that is, verification of *non-interference*.

**Case Study 4: Fairness in non-repudiation protocols.** A *non-repudiation* protocol ensures that a receiver obtains a receipt from the sender, called *non-repudiation of origin* (*NRO*), and the sender ends up having an evidence, named *non-repudiation of receipt* (*NRR*), through a trusted third party. A non-repudiation protocol is *fair* if both *NRR* and *NRO* are either received or not received by the parties (see formula $\varphi_{\mathsf{fair}}$ in Table 2). We verified two different protocols from [31], namely, $T_{incorrect}$ that chooses not to send out *NRR* after receiving *NRO*, and a correct implementation $T_{correct}$ which is fair. For $T_{correct}$

(respectively, $T_{incorrect}$), HyperQube returns UNSAT in the halting optimistic semantics (respectively, SAT in the halting pessimistic semantics), which indicates that the protocol satisfies (respectively, violates) fairness.

**Case Study 5: Path planning for robots.** We have used HyperQube beyond verification, to synthesize strategies for robotic planning [34]. Here, we focus on producing a strategy that satisfies two control requirements for a robot to reach a goal in a grid. First, the robot should take the *shortest path* (see formula $\varphi_{sp}$ in Table 2). Fig. 2 shows a $10 \times 10$ grid, where the red, green, and black cells are initial, goal, and blocked cells, respectively. HyperQube returns SAT and the synthesized path is shown by the blue arrows. We also used HyperQube to solve the *path robustness* problem, meaning that starting from an arbitrary initial state, a robot reaches the goal by following a single strategy (see formula $\varphi_{rb}$ in Table 2). Again, HyperQube returns SAT for the grid shown in Fig. 3.

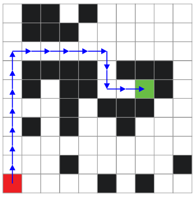

**Fig. 2:** Shortest Path

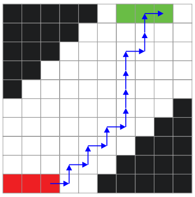

**Fig. 3:** Robust path

**Case Study 6: Mutation testing.** We adopted the model from [15] and apply the original formula that describes a good test mutant together with the model (see formula $\varphi_{\mathsf{mut}}$ in Table 2). HyperQube returns SAT, indicating successful finding of a qualified mutant. We note that in [15] the authors were not able to generate test cases via $\varphi_{\mathsf{mut}}$, as the model checker MCHyper is not able to handle quantifier alternation in push-button fashion.

**Results and analysis.** Table 3 summarizes our results including running times, the bounded semantics applied, the output of the QBF solver, and the resulting infinite inference conclusion using Theorem 1. As can be seen, our case studies range over model checking of different fragments of HyperLTL. It is important to note that HyperQube run time consists of generating a QBF formula by genqbf and then checking its satisfiability by QuAbS. It is remarkable that in some cases, QBF formula generation takes longer than checking its satisfiability. The models in our experiments also have different sizes. The most complex case study is arguably the SNARK algorithm, where we identify both bugs in the algorithm in 472 and 1497 seconds. In cases 5.1 – 6.2, we also demonstrate the ability of HyperQube to solve synthesis problems by leveraging the existential quantifier in a HyperLTL formula.

Finally, we elaborate more on scalability of the path planning problem for robots. This problem was first studied in [34], where the authors reduce the problem to SMT solving using Z3 [13] and by eliminating the trace quantifiers through a combinatorial enumeration of conjunctions and disjunctions. Table 4 compares our approach with the brute-force technique employed in [34] for different grid sizes. Our QBF-based approach clearly outperforms the solution in [34], in some cases by an order of magnitude.

| # | Model $K$ | Formula | bound $k$ | $\|AP^*\|$ | QBF | semantics | genqbf [s] | QuAbS [s] | **Total** [s] | |
|---|---|---|---|---|---|---|---|---|---|---|
| 0.1 | Bakery.3proc | $\varphi_{S1}$ | 7 | 27 | SAT | *pes* | 0.44 | 0.04 | **0.48** | ✗ |
| 0.2 | Bakery.3proc | $\varphi_{S2}$ | 12 | 27 | SAT | *pes* | 1.31 | 0.15 | **1.46** | ✗ |
| 0.3 | Bakery.3proc | $\varphi_{S3}$ | 20 | 27 | UNSAT | *opt* | 2.86 | 4.87 | **7.73** | ✓ |
| 1.1 | Bakery.3proc | $\varphi_{sym1}$ | 10 | 27 | SAT | *pes* | 0.86 | 0.11 | **0.97** | ✗ |
| 1.2 | Bakery.3proc | $\varphi_{sym2}$ | 10 | 27 | SAT | *pes* | 0.76 | 0.17 | **0.93** | ✗ |
| 1.3 | Bakery.5proc | $\varphi_{sym1}$ | 10 | 45 | SAT | *pes* | 23.57 | 1.08 | **24.65** | ✗ |
| 1.4 | Bakery.5proc | $\varphi_{sym2}$ | 10 | 45 | SAT | *pes* | 29.92 | 1.43 | **31.35** | ✗ |
| 2.1 | SNARK-bug1 | $\varphi_{lin}$ | 26 | 160 | SAT | *pes* | 88.42 | 383.60 | **472.02** | ✗ |
| 2.2 | SNARK-bug2 | $\varphi_{lin}$ | 40 | 160 | SAT | *pes* | 718.09 | 779.76 | **1497.85** | ✗ |
| 3.1 | 3-$Thread_{incorrect}$ | $\varphi_{NI}$ | 57 | 31 | SAT | *h-pes* | 19.56 | 46.66 | **66.22** | ✗ |
| 3.2 | 3-$Thread_{correct}$ | $\varphi_{NI}$ | 57 | 31 | UNSAT | *h-opt* | 23.91 | 33.54 | **57.45** | ✓ |
| 4.1 | $NRP : T_{incorrect}$ | $\varphi_{fair}$ | 15 | 15 | SAT | *h-pes* | 0.10 | 0.27 | **0.37** | ✗ |
| 4.2 | $NRP : T_{correct}$ | $\varphi_{fair}$ | 15 | 15 | UNSAT | *h-opt* | 0.08 | 0.12 | **0.20** | ✓ |
| 5.1 | Shortest Path | (see Table 4) | | | | | | | | synthesis |
| 5.2 | Initial State Robustness | | | | | | | | | |
| 6.1 | Mutant | $\varphi_{mut}$ | 8 | 6 | SAT | *h-pes* | 1.40 | 0.35 | **1.75** | |

**Table 3:** Performance of HyperQube, where column *case#* identifies the artifact, ✓ denotes satisfaction, and ✗ denotes violation of the formula. $AP^*$ is the set of Boolean variables encoding $K$.

| | | | HyperQube | | | | [34] | | |
|---|---|---|---|---|---|---|---|---|---|
| Formula | grid size | bound $k$ | $\|AP^*\|$ | genqbf [s] | QuAbS [s] | **Total [s]** | gensmt [s] | Z3 [s] | **Total[s]** |
| $\varphi_{sp}$ | $10^2$ | 20 | 12 | 1.30 | 0.57 | **1.87** | 8.31 | 0.33 | **8.64** |
| | $20^2$ | 40 | 14 | 4.53 | 12.16 | **16.69** | 124.66 | 6.41 | **131.06** |
| | $40^2$ | 80 | 16 | 36.04 | 35.75 | **71.79** | 1093.12 | 72.99 | **1166.11** |
| | $60^2$ | 120 | 16 | 105.82 | 120.84 | **226.66** | 4360.75 | 532.11 | **4892.86** |
| $\varphi_{rb}$ | $10^2$ | 20 | 12 | 1.40 | 0.35 | **1.75** | 11.14 | 0.45 | **11.59** |
| | $20^2$ | 40 | 14 | 15.92 | 15.32 | **31.14** | 49.59 | 2.67 | **52.26** |
| | $40^2$ | 80 | 16 | 63.16 | 20.13 | **83.29** | 216.16 | 19.81 | **235.97** |

**Table 4:** Path planning for robots and comparison to [34]. All cases use the halting pessimistic semantics and QBF solver returns SAT, meaning successful path synthesis.

## 6 Related Work

There has been a lot of recent progress in automatically verifying [12,22–24] and monitoring [1,6,7,20,21,26,33] HyperLTL specifications. HyperLTL is also supported by a growing set of tools, including the model checker MCHyper [12,24], the satisfiability checkers EAHyper [19] and MGHyper [17], and the runtime monitoring tool RVHyper [20]. The complexity of *model checking* for HyperLTL for tree-shaped, acyclic, and general graphs was rigorously investigated in [2]. The first algorithms for model checking HyperLTL and HyperCTL* using alternating automata were introduced in [24]. These techniques, however, were not able to deal in practice with alternating HyperLTL formulas in a fully automated fashion. We also note that previous approaches that reduce model checking HyperLTL—typically of formulas without quantifier alternations—to model checking LTL can use BMC in the LTL model checking phase. However, this is a different approach than the one presented here, as these approaches simply instruct the model checker to use a BMC *after* the problem has been fully reduced to an LTL model checking problem while we avoid this translation. These algorithms were then extended to deal with hyperliveness and alternating formulas in [12] by finding a winning strategy in $\forall\exists$ games. In this paper, we take an alternative approach by reducing the model checking problem to QBF solving, which is arguably more effective for finding bugs (in case a finite witness exists).

The *satisfiability* problem for HyperLTL is shown to be undecidable in general but decidable for the $\exists^*\forall^*$ fragment and for any fragment that includes a $\forall\exists$ quantifier alternation [16]. The hierarchy of hyperlogics beyond HyperLTL were studied in [11]. The synthesis problem for HyperLTL has been studied in [3] in the form of *program repair*, in [4] in the form of *controller synthesis*, and in [18] for the general case.

## 7 Conclusion and Future Work

We introduced the first bounded model checking (BMC) technique for verification of hyperproperties expressed in HyperLTL. To this end, we proposed four different semantics that ensure the soundness of inferring the outcome of the model checking problem. To handle trace quantification in HyperLTL, we reduced the BMC problem to checking satisfiability of quantified Boolean formulas (QBF). This is analogous to the reduction of BMC for LTL to the simple propositional satisfiability problem. We have introduced different classes of semantics, beyond the pessimistic semantics common in LTL model checking, namely *optimistic* semantics that allow to infer full verification by observing only a finite prefix and *halting* variations of these semantics that additionally exploit the termination of the execution, when available. Through a rich set of case studies, we demonstrated the effectiveness and efficiency of our approach in verification of information-flow properties, linearizability in concurrent data structures, path planning in robotics, and fairness in non-repudiation protocols.

As for future work, our first step is to solve the loop condition problem. This is necessary to establish completeness conditions for BMC and can help cover even more examples efficiently. The application of QBF-based techniques in the framework of abstraction/refinement is another unexplored area. Success of BMC for hyperproperties inherently depends on effectiveness of QBF solvers. Even though QBF solving is not as mature as SAT/SMT solving techniques, recent breakthroughs on QBF have enabled the construction of our tool HyperQube, and more progress in QBF solving will improve its efficiency.

## References

[1] Shreya Agrawal and Borzoo Bonakdarpour. Runtime verification of $$k$$-safety hyperproperties in HyperLTL. In *Proc. of the 29th IEEE Computer Security Foundations Symposium (CSF’16)*, pages 239–252. IEEE, 2016.

[2] Borzoo Bonakdarpour and Bernd Finkbeiner. The complexity of monitoring hyperproperties. In *Proc. of the IEEE 31st Computer Security Foundations Symposium (CSF’18)*, pages 162–174. IEEE, 2018.

[3] Borzoo Bonakdarpour and Bernd Finkbeiner. Program repair for hyperproperties. In *Proc. of the 17th Symposium on Automated Technology for Verification and Analysis (ATVA’19)*, volume 11781 of *LNCS*, pages 423–441. Springer, 2019.

[4] Borzoo Bonakdarpour and Bernd Finkbeiner. Controller synthesis for hyperproperties. In *Proc. of the 33rd IEEE Computer Security Foundations Symposium (CSF’20)*, pages 366–379. IEEE, 2020.

[5] Borzoo Bonakdarpour, Pavithra Prabhakar, and César Sánchez. Model checking timed hyperproperties in discrete-time systems. In *Proc. of the 12th NASA Formal Methods Symposium (NFM’20)*, volume 12229 of *LNCS*, pages 311–328. Springer, 2020.

[6] Borzoo Bonakdarpour, César Sánchez, and Gerardo Schneider. Monitoring hyperproperties by combining static analysis and runtime verification. In *Proc. of the 8th Int’l Symposium on Leveraging Applications of Formal Methods, Verification and Validation (ISoLA’18), Part II*, volume 11245 of *LNCS*, pages 8–27. Springer, 2018.

[7] Noel Brett, Umair Siddique, and Borzoo Bonakdarpour. Rewriting-based runtime verification for alternation-free HyperLTL. In *Proc. of the 23rd Int’l Conf. on Tools and Algorithms for the Construction and Analysis of Systems (TACAS’17), Part II*, volume 10206 of *LNCS*, pages 77–93. Springer, 2017.

[8] Edmund M. Clarke, Armin Biere, Richard Raimi, and Yunshan Zhu. Bounded model checking using satisfiability solving. *Formal Methods in System Design*, 19(1):7–34, 2001.

[9] Michael R. Clarkson, Bernd Finkbeiner, Masoud Koleini, Kristopher K. Micinski, Markus N. Rabe, and César Sánchez. Temporal logics for hyperproperties. In *Proc. of the 3rd Int’l Conf. on Principles of Security and Trust (POST’14)*, volume 8414 of *LNCS*, pages 265–284. Springer, 2014.

[10] Michael R. Clarkson and Fred B. Schneider. Hyperproperties. *Journal of Computer Security*, 18(6):1157–1210, 2010.

[11] Norine Coenen, Bernd Finkbeiner, Cristopher Hahn, and Jana Hofmann. The hierarchy of hyperlogics. In *Proc. of the 34th Annual ACM/IEEE Symposium on Logic in Computer Science (LICS’19)*, pages 1–13. IEEE, 2019.

[12] Norine Coenen, Bernd Finkbeiner, César Sánchez, and Leander Tentrup. Verifying hyperliveness. In *Proc. of the 31st Int’l Conf. on Computer Aided Verification (CAV’19), Part I*, volume 11561 of *LNCS*, pages 121–139. Springer, 2019.

[13] Leonardo de Moura and Nikolaj Bjorner. Z3 – a tutorial. Technical report, Microsoft, 2012.

[14] Simon Doherty, David Detlefs, Lindsay Groves, Christine H. Flood, Victor Luchangco, Paul Alan Martin, Mark Moir, Nir Shavit, and Guy L. Steele Jr. DCAS is not a silver bullet for nonblocking algorithm design. In *Proc. of the 16th Annual ACM Symposium on Parallelism in Algorithms and Architectures (SPAA’04)*, pages 216–224. ACM, 2004.

[15] Andreas Fellner, Mitra Tabaei Befrouei, and Georg Weissenbacher. Mutation testing with hyperproperties. In *Proc. of the 17th Int’l Conf. on Software Engineering and Formal Methods (SEFM’19)*, volume 11724 of *LNCS*, pages 203–221. Springer, 2019.

[16] Bernd Finkbeiner and Cristopher Hahn. Deciding hyperproperties. In *Proc. of the 27th Int’l Conf. on Concurrency Theory (CONCUR’16)*, volume 59 of *LIPIcs*, pages 13:1–13:14. Schloss Dagstuhl - Leibniz-Zentrum für Informatik, 2016.

[17] Bernd Finkbeiner, Cristopher Hahn, and Tobias Hans. MGHyper: Checking satisfiability of HyperLTL formulas beyond the $$\exists ^*\forall ^*$$ fragment. In *Proc. of the 16th Int’l Symposium on Automated Technology for Verification and Analysis (ATVA’18)*, volume 11138 of *LNCS*, pages 521–527. Springer, 2018.

[18] Bernd Finkbeiner, Cristopher Hahn, Philip Lukert, Marvin Stenger, and Leander Tentrup. Synthesis from hyperproperties. *Acta Informatica*, 57(1-2):137–163, 2020. 10.1007/s00236-019-00358-2 PMC7056710 32189717

[19] Bernd Finkbeiner, Cristopher Hahn, and Marvin Stenger. Eahyper: Satisfiability, implication, and equivalence checking of hyperproperties. In *Proc. of the 29th Int’l Conf. on Computer Aided Verification (CAV’17), Part II*, volume 10427 of *LNCS*, pages 564–570. Springer, 2017.

[20] Bernd Finkbeiner, Cristopher Hahn, Marvin Stenger, and Leander Tentrup. RVHyper: A runtime verification tool for temporal hyperproperties. In *Proc. of the 24th Int’l Conf. on Tools and Algorithms for the Construction and Analysis of Systems (TACAS’18), Part II*, volume 10806 of *LNCS*, pages 194–200. Springer, 2018.

[21] Bernd Finkbeiner, Cristopher Hahn, Marvin Stenger, and Leander Tentrup. Monitoring hyperproperties. *Formal Methods in System Design*, 54(3):336–363, 2019. 10.1007/s10703-019-00334-z PMC6853877 31806925

[22] Bernd Finkbeiner, Cristopher Hahn, and Hazem Torfah. Model checking quantitative hyperproperties. In *Proc. of the 30th Int’l Conf. on Computer Aided Verification (CAV’18), Part I*, volume 10981 of *LNCS*, pages 144–163. Springer, 2018.

[23] Bernd Finkbeiner, Christian Müller, Helmut Seidl, and Eugene Zalinescu. Verifying security policies in multi-agent workflows with loops. In *Proc. of the 15th ACM Conf. on Computer and Communications Security (CCS’17)*, pages 633–645. ACM, 2017.

[24] Bernd Finkbeiner, Markus N. Rabe, and César Sánchez. Algorithms for model checking HyperLTL and HyperCTL*. In *Proc. of the 27th Int’l Conf. on Computer Aided Verification (CAV’15), Part I*, volume 9206 of *LNCS*, pages 30–48. Springer, 2015.

[25] Joseph A. Goguen and José Meseguer. Security policies and security models. In *1982 IEEE Symposium on Security and Privacy*, pages 11–20. IEEE Computer Society, 1982.

[26] Cristopher Hahn, Marvin Stenger, and Leander Tentrup. Constraint-based monitoring of hyperproperties. In *Proc. of the 25th Int’l Conf. on Tools and Algorithms for the Construction and Analysis of Systems (TACAS’19)*, volume 11428 of *LNCS*, pages 115–131. Springer, 2019.

[27] Klaus Havelund and Doron Peled. Runtime verification: From propositional to first-order temporal logic. In *Proc. of the 18th Int’l Conf. on Runtime Verification (RV’18)*, volume 11237 of *LNCS*, pages 90–112. Springer, 2018.

[28] Jesko Hecking-Harbusch and Leander Tentrup. Solving QBF by abstraction. In *Proc. of the 9th Int’l Symposium on Games, Automata, Logics and Formal Verification (GandALF’18)*, volume 277 of *EPTCS*, pages 88–102, 2018.

[29] Maurice Herlihy and Jeannette M. Wing. Linearizability: A correctness condition for concurrent objects. *ACM Transactions on Programming Languages and Systems*, 12(3):463–492, 1990.

[30] Tzu-Han Hsu, César Sánchez, and Borzoo Bonakdarpour. Bounded model checking for hyperproperties. *CoRR*, abs/2009.08907, 2020.

[31] Wojciech Jamroga, Sjouke Mauw, and Matthijs Melissen. Fairness in non-repudiation protocols. In *Proc. of the 7th Int’l Workshop on Security and Trust Management (STM’11)*, volume 7170 of *LNCS*, pages 122–139. Springer, 2011.

[32] Geoffrey Smith and Dennis M. Volpano. Secure information flow in a multi-threaded imperative language. In *Proc. of the 25th ACM Symposium on Principles of Programming Languages (POPL’98)*, pages 355–364. ACM, 1998.

[33] Sandro Stucki, César Sánchez, Gerardo Schneider, and Borzoo Bonakdarpour. Graybox monitoring of hyperproperties. In *Proc. of the 23rd Int’l Symposium on Formal Methods (FM’19)*, volume 11800 of *LNCS*, pages 406–424. Springer, 2019.

[34] Yu Wang, Siddharta Nalluri, and Miroslav Pajic. Hyperproperties for robotics: Planning via HyperLTL. In 2020 IEEE Int’l Conf. on Robotics and Automation (ICRA’20), pages 8011–8017. IEEE, 2020.

[35] Yu Wang, Mojtaba Zarei, Borzoo Bonakdarpour, and Miroslav Pajic. Statistical verification of hyperproperties for cyber-physical systems. *ACM Transactions on Embedded Computing systems*, 18(5s):92:1–92:23, 2019.

